# Sex Differences in the Global Prevalence of Nonsuicidal Self-Injury in Adolescents: A Meta-Analysis

**DOI:** 10.1001/jamanetworkopen.2024.15436

**Published:** 2024-06-14

**Authors:** Fiona Moloney, Jasmine Amini, Mark Sinyor, Ayal Schaffer, Krista L. Lanctôt, Rachel H.B. Mitchell

**Affiliations:** 1 Department of Psychiatry, University of Toronto, Toronto, Ontario, Canada; 2 Department of Psychiatry, Sunnybrook Health Sciences Centre, Toronto, Ontario, Canada

## Abstract

**Importance:**

Nonsuicidal self-injury (NSSI) is a strong predictor of suicide attempts. The prevalence of NSSI has been increasing among female adolescents in North America and Europe, but less is known about trends in other geographical regions.

**Objective:**

To examine sex differences in the prevalence of NSSI among adolescents within and between geographical regions.

**Data Sources:**

MEDLINE and PsycINFO were searched using the keywords *adolescents*, *self-injury*, *sex factors*, and synonyms for articles published in English between January 1, 2000, and May 10, 2022.

**Study Selection:**

Studies were included if they presented original data (any study design), included adolescents aged 10 to 19 years, reported results stratified by sex, and explicitly defined self-injury as behaviors occurring without suicidal intent.

**Data Extraction and Synthesis:**

This meta-analysis was registered with PROSPERO and conducted in accordance with the Preferred Reporting Items for Systematic Reviews and Meta-Analyses. Articles were assessed for quality by 2 independent coders (F.M. and J.A.). A random-effects model was used to calculate prevalence. Data were analyzed from July 2022 to April 2023.

**Main Outcomes and Measures:**

The prevalence of NSSI in male and female adolescents within and between regions was the main outcome. Odds ratios (OR) with 95% CIs were calculated for community samples.

**Results:**

Eight hundred and two studies were screened, and 38 were included (266 491 participants). Across 17 countries, the pooled prevalence of NSSI was 17.7% (female:male OR, 1.60; 95% CI, 1.29-1.98). NSSI was twice as prevalent among female adolescents compared with male adolescents in North America (OR, 2.49; 95% CI, 2.16-2.86) and Europe (OR, 2.08; 95% CI, 1.69-2.58), but not in Asia (OR, 1.00; 95% CI, 0.71-1.41).

**Conclusions and Relevance:**

In this meta-analysis of sex differences in global prevalence of NSSI, the female predominance of NSSI observed among adolescents in North America and Europe aligned with rising rates of suicide in these populations. The comparable prevalence of NSSI among male and female adolescents in Asia also aligned with the lower male-to-female suicide ratio compared with other countries. More research is needed to characterize regional (and potentially cultural) sex differences among adolescents with NSSI to prevent and treat the behavior and to understand the possible interplay with corresponding regional trends in suicide.

## Key Points

**Question:**

Does the prevalence of nonsuicidal self-injury (NSSI) among female adolescents and male adolescents vary by geography?

**Findings:**

This meta-analysis of 38 studies with 266 491 participants found that NSSI was twice as prevalent among female adolescents vs male adolescents in North America and Europe, but not in Asia. The study also found a higher prevalence of NSSI among male adolescents in Asia compared with other regions.

**Meaning:**

It is important to understand why NSSI may be more prevalent among female adolescents compared with male adolescents in some regions, but not in others, to effectively prevent and treat the behavior among all adolescents.

## Teaser

This meta-analysis evaluates sex differences in the prevalence of nonsuicidal self-injury among adolescents within and between geographical regions.

## Introduction

Nonsuicidal self-injury (NSSI) is defined as “the deliberate, self-inflicted destruction of body tissue resulting in immediate damage, without suicidal intent and for purposes not culturally sanctioned.”^1^ The mean age of onset for NSSI is approximately 13 years,^2^ and the behavior is common among adolescents,^3,4,5^ with global prevalence estimates between 18% and 23%.^1,2,6^ Historically, there has been debate on the validity, and therefore merit, of distinguishing suicide-related behavior as with or without suicidal intent.^7^ However, it is now generally understood that any self-harm or self-injurious behavior among adolescents, regardless of suicidal intent (and including NSSI), is a robust predictor of death by suicide.^8,9,10,11,12^ Moreover, NSSI as a phenotype has been studied among youths across the world,^2^ and knowledge of this behavior in a global context is especially important because of the associated risk of suicide.

NSSI is often reported as more prevalent in female vs male adolescents,^3,4,5,13,14,15,16,17,18,19,20,21,22,23,24,25,26,27,28,29,30,31,32,33,34,35,36^ although some studies report no sex difference^1,37,38^ or higher prevalence among male adolescents.^39,40,41,42,43,44,45^ In North America, the rates of NSSI are rising among all adolescents,^46^ but disproportionately among younger female adolescents.^47,48^ There is also preliminary evidence to suggest that the prevalence of NSSI among adolescents differs by sex across geographic regions.^38^ To date, studies reporting prevalence of NSSI among adolescents tend to present the findings disaggregated by sex; however, a systematic approach is needed to evaluate and quantify the geographic variability in the prevalence of NSSI among female vs male adolescents worldwide. This information is essential to develop sex-, regional-, and potentially culture-specific interventions for NSSI among adolescents across the globe that can ultimately mitigate the risk of suicide in these populations. Therefore, the objective of this study is to examine sex differences in the global and regional prevalence of NSSI among adolescents (henceforth defined as those aged 10 to 19 years).

## Methods

A systematic review of the literature on sex differences in the prevalence of adolescents with NSSI was performed according to the Preferred Reporting Items for Systematic Reviews and Meta-analyses (PRISMA) reporting guideline.^49^ The protocol for this review was registered with the international prospective register of systematic reviews before data extraction and analysis (CRD42022347504). This protocol describes a systematic review that provided data for 2 studies: the current meta-analysis and a separate systematic review of sex differences in the clinical correlates of NSSI among adolescents.

### Search Strategy

We searched MEDLINE and APA PsycINFO with the terms *adolescents*, *self-injury*, *sex factors*, and synonyms in May 2022. A complete search strategy can be found in the eMethods in Supplement 1.

### Inclusion Criteria

Studies were included if they were published in English and reported on NSSI in female and male adolescents aged 10 to 19 years. Studies were only included if they reported original data and explicitly defined self-harm as not having suicidal intent. Given the paucity of research on sex differences in NSSI, studies were not excluded on the basis of study design. Similarly, studies were included regardless of whether they defined a specific period during which the NSSI occurred (eg, recent or lifetime occurrence). The reference lists of included articles were also reviewed for relevant citations.

### Exclusion Criteria

**Figure 1.**
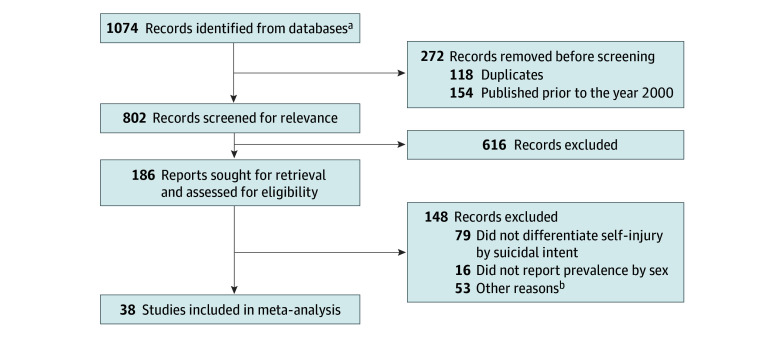
Flow Diagram of the Study Selection Process ^a^Eight hundred fifty-three from MEDLINE, 221 from APA PsycINFO. ^b^Excluded due to limited relevance (eg, only reported on NSSI in individuals with 1 specific diagnosis or characteristic).

Studies were preliminarily excluded if the data for adolescents were not reported separately from other age groups, if they did not clearly differentiate self-injury based on suicidal intent, or if they did not report on prevalence in female and male adolescents separately. Articles were also excluded if they had limited relevance to the topic of this meta-analysis, including only reporting on NSSI in individuals with a specific diagnosis or characteristic. Grey literature and conference abstracts were also excluded. Articles published before January 2000 were excluded, as the early 2000s marked a turning point in research in the area, in part due to the creation of the first validated measure for what was then called deliberate self-harm (including NSSI).^50^ Lastly, all eligible articles were quality assessed by 2 independent reviewers (F.M. and J.A.) using the Joanna Briggs Institute Critical Appraisal Checklist for Cross-Sectional Studies^51^ or the Joanna Briggs Institute Critical Appraisal Checklist for Cohort Studies.^52^ Articles were excluded if they received less than 4 on their respective checklists. The article selection process is shown in Figure 1.

### Article Selection Process

Articles were screened by title and abstract by 2 independent unblinded reviewers (F.M. and R.H.B.M.). Full-text reports were reviewed for study eligibility by the same reviewers. Discrepancies were resolved by consensus.

### Data Collection

Data were recorded for the following categories: (1) author(s); (2) year of publication; (3) country of origin (where the study was published or conducted); (4) aims or purpose; (5) population and sample size; (6) methods. For characteristics of studies included, please refer to eTable 1 in Supplement 1. Geographical regions were defined by continent.

### Statistical Analysis

Prevalence data were extracted and quantitatively pooled. Random-effects meta-analysis was conducted using Meta-Essentials version 1.5 for Microsoft Excel (Erasmus Research Institute of Management).^53^ A random-effects model was chosen given the high expected heterogeneity of included studies. Odds ratios (OR) were used as the indicator of effect size. The weighting method used was inverse variance. ORs were calculated for community samples only, as clinical samples are less generalizable to the overall population. Post hoc analyses were conducted using 2-way analyses of variance (ANOVAs) to assess regional variation of NSSI prevalence within sex.

Heterogeneity was assessed using the *I^2^* statistic. For analyses with an *I^2^* greater than 75%, which generally indicates that a large proportion of variability can be accounted for by heterogeneity between studies, moderator analyses were conducted. Moderator analyses were run for year of publication (mean-centered on 2015), survey time period (lifetime NSSI vs past month to 5 years), and geographical region (Europe vs Asia and North America and Asia vs Europe and North America) separately using regression analyses to attempt to determine sources of heterogeneity. These moderators were chosen given that trends in suicide-related behavior change over time, that studies looking only at recent NSSI may show different characteristics than those looking at lifetime occurrences, and that certain regions may show greater differences in reporting and analyzing NSSI than others, respectively. Presence of publication bias was assessed with visual inspection of funnel plots and Egger regression. The threshold for statistical significance was *P* = .05, using 2-sided tests for statistical analyses. Data were analyzed from July 2022 to April 2023.

## Results

**Figure 2.**
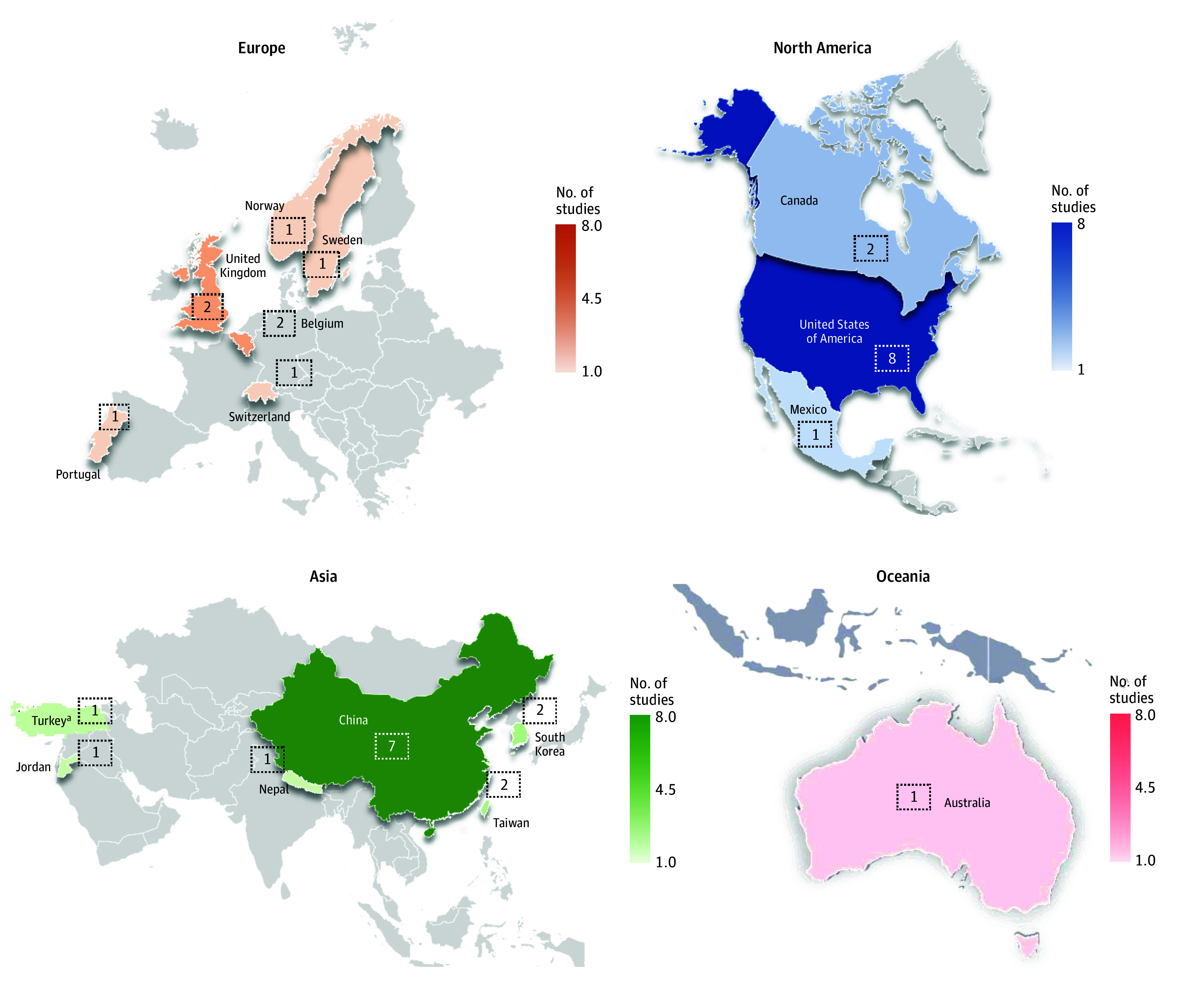
Geographical Distribution of Included Community Samples, Separated by Continent Number in box indicates No. of studies from each region. ^a^Turkey is part of both Europe and Asia. For the purposes of this study, Turkey was included as a country in Asia.

Thirty-eight articles met inclusion criteria comprising a total of 266 491 participants across 17 countries. Of the unique samples, 34 were community samples^3,4,5,13,14,17,18,20,21,22,23,24,25,26,27,30,31,32,33,34,35,37,38,40,41,42,43,44,45,54,55,56,57,58^ and 4 were clinical.^16,28,29,59^ The regional breakdown of community samples consisted of 11 from North America (1 from Mexico, 2 from Canada, and 8 from the US), 14 from Asia (1 from Jordan, 1 from Turkey, 7 from China, 2 from Taiwan, 2 from South Korea, and 1 from Nepal), 8 from Europe (1 from Switzerland, 1 from Sweden, 2 from Belgium, 2 from UK, 1 from Norway, and 1 from Portugal), and 1 from Australia (Figure 2). The mean (range) quality index score for cross-sectional studies was 6.3 (4-8) and for cohort studies was 9.0 (8-10).

### Prevalence

**Figure 3.**
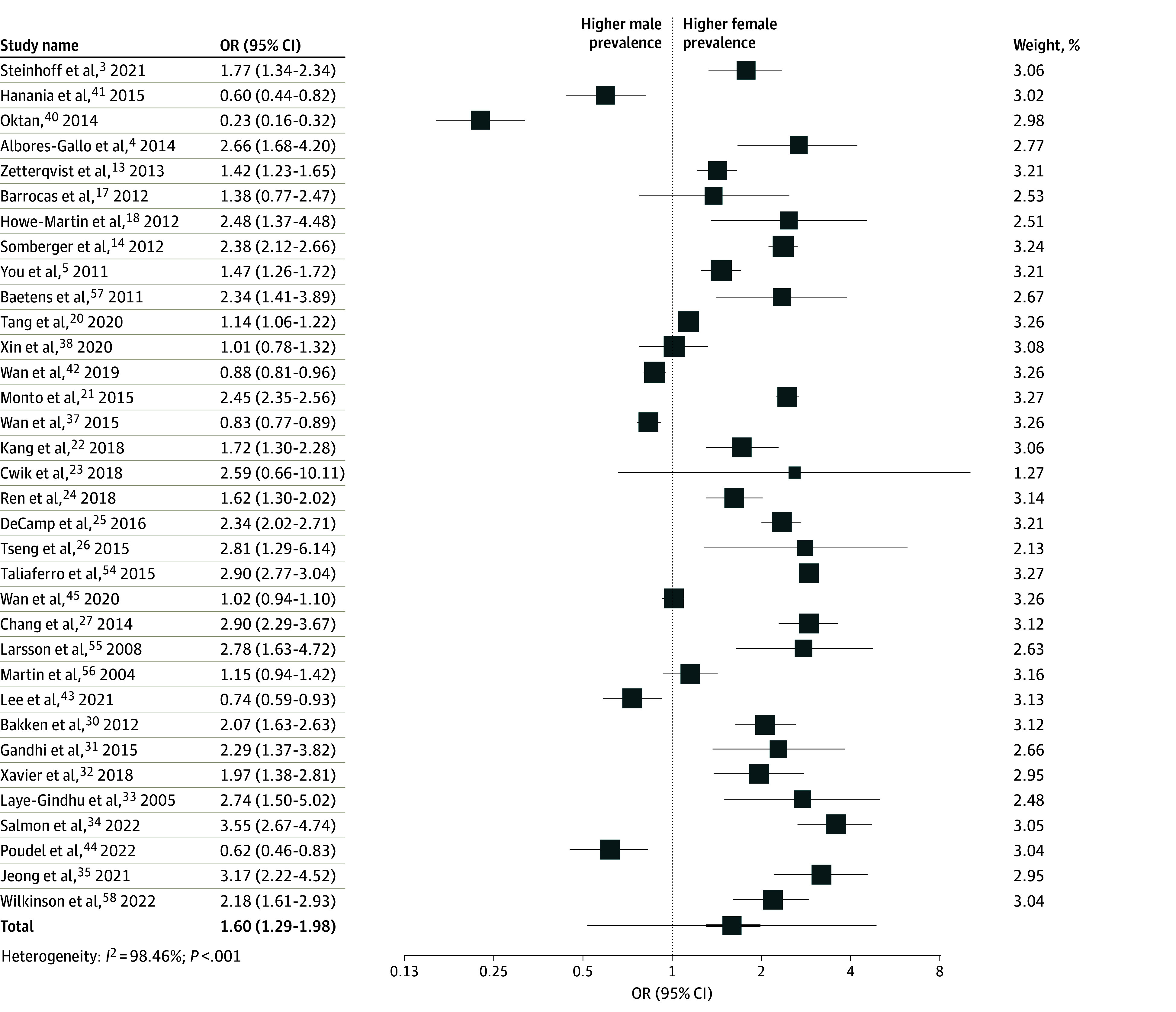
Forest Plot Depicting Odds Ratio (OR; Effect Size) for Nonsuicidal Self-Injury in Female Adolescents as Compared With Male Adolescents for All Included Community Studies Total row indicates overall effect size with CI (bold) and prediction interval (interval within which future value will fall).

The pooled prevalence of NSSI by sex (38 studies, 266 491 participants) was 21.4% for female adolescents (29 161 participants; 95% CI, 21.2%-21.7%) and 13.7% for male adolescents (17 907 participants; 95% CI, 13.5%-13.9%). Across 17 countries, the pooled prevalence of NSSI in community samples was 17.6% (95% CI, 17.5%-17.7%). The pooled prevalence in community samples (34 studies; 263 678 participants) was 21.3% (95% CI, 21.1%-21.5%) for female adolescents and 13.7% (95% CI, 13.5%-13.9%) for male adolescents. The pooled prevalence in clinical samples (4 studies; 2813 participants) was 34.9% (95% CI, 32.3%-37.4%) for female adolescents and 13.6% (95% CI, 11.8%-15.2%) for male adolescents. The female-to-male OR for community prevalence of NSSI was 1.60 (95% CI, 1.29%-1.98) (Figure 3).

There was substantial heterogeneity (*Q* = 2147; *P* < .001; *I^2^* = 98.46%); as such, moderator analyses were completed. A more recent publication year (as compared with the year 2015) (β = −0.30; 95% CI, −0.06 to −0.04; *P* < .001) and the geographical region of Asia (vs North America and Europe combined) were associated with a smaller effect size (β = −0.90; 95% CI, −0.98 to −0.89; *P* < .001), whereas studies assessing lifetime NSSI (vs NSSI within the prior 5 years) were associated with a larger effect size (β = 0.43; 95% CI, 0.43 to 0.52; *P* < .001). The geographical region of Europe (vs North America and Asia combined) (β = 0.03; 95% CI, −0.02 to 0.18; *P* = .12) was not significantly associated with effect size. See eFigure 1, eFigure 2, eFigure 3, and eFigure 4 in Supplement 1 for the complete results of the moderator analysis.

Publication bias (see eFigure 5 in Supplement 1) did not reveal any obvious asymmetry on visual inspection, with the exception of 1 outlier. Egger regression was also not statistically significant (*t* = −1.08; *P* = .29), indicating that publication bias is unlikely to have significantly affected the findings of this analysis.

### Prevalence by Region

**Figure 4.**
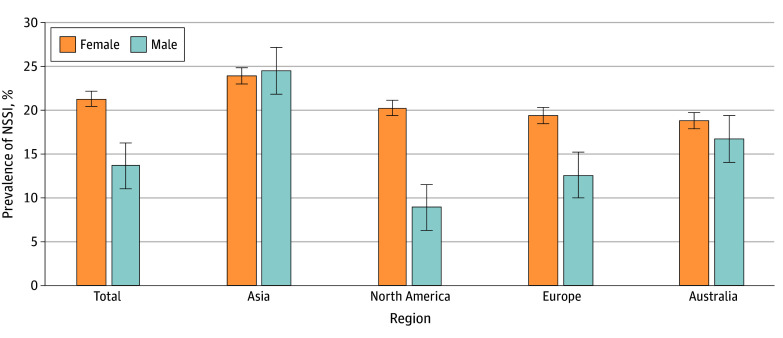
Pooled Prevalence of Nonsuicidal Self-Injury (NSSI) by Gender and Region (Community Samples Only) Error bars denote SE.

The community prevalence of NSSI among adolescents was significantly higher in female adolescents than male adolescents in North America (20.2% and 8.9% respectively; OR, 2.49; 95% CI, 2.16-2.86) and Europe (19.4% and 12.6% respectively; OR, 2.08; 95% CI, 1.69-2.58) (Figure 3). It was also higher in the single Australian sample (18.8% and 16.8%, respectively; OR, 1.15). In contrast, prevalence rates were not significantly different between sexes in Asia (24.1% for female adolescents vs 24.8% for male adolescents; OR, 1.00; 95% CI, 0.71-1.41) (Figure 4). All 19 studies in North America or Europe reported higher prevalence of NSSI in female adolescents; however, 8 of 14 studies in Asia reported equal or higher prevalence of NSSI in male adolescents.^37,38,40,41,42,43,44,45^

Post hoc analyses using ANOVA did not reveal a regional difference in the prevalence of NSSI among female adolescents (*F*_2,30_ = 5.31; *P* = .01). In contrast, there was some regional variation in the prevalence of NSSI among male adolescents (*P* = .01), with higher prevalence reported among male adolescents in Asia compared with North America (*t*_17_ = 3.15; *P* = .003) and Europe (*t*_20_ = 2.29; *P* = .02). There was no regional variation in the prevalence of NSSI among male adolescents in Europe as compared with North America (*t*_10_ = 0.45; *P* = .33). ANOVA and *t* test statistics can be found in eTable 2 in Supplement 1.

## Discussion

This meta-analysis summarizes sex differences in the prevalence of NSSI among adolescents around the world. We report a higher prevalence of NSSI among female adolescents compared with male adolescents overall, although considerable regional variation exists. In North America, Europe, and Australia, the prevalence of NSSI was higher among female adolescents; whereas in Asia, the prevalence of NSSI did not significantly differ by sex, and in some samples was higher in male adolescents. There were no regional differences in the prevalence of NSSI in female adolescents; however, male adolescents in Asia had a higher prevalence of NSSI than male adolescents in other regions.

To date, the literature on sex differences in the prevalence of NSSI among adolescents has reported inconsistent results, with some studies recording higher prevalence in female adolescents and others recording no sex difference.^1,2^ This meta-analysis reports a higher overall prevalence of NSSI among all pooled female adolescents compared with male adolescents, with an OR of 1.60, denoting a moderate effect size.^60^ This higher prevalence of NSSI among female adolescents builds on findings from a recent meta-analysis^2^ of 158 samples showing female adolescents report significantly more self-harm than male adolescents, with an OR of 1.72 (ie, moderate effect size), although direct comparisons are not possible as Gillies et al^2^ use a broader definition of self-harm that does not specify suicidal intent. While the large proportion of Western samples in both meta-analyses likely influences the effect sizes reported, it is nevertheless worth considering if higher rates of NSSI in female adolescents across the globe reflect known sex differences in depressive disorders, internalizing symptoms, and identity disturbance that are more common in female adolescents compared with male adolescents, and if these factors moderate the relationship between NSSI and female sex, regardless of the region of the world.^31,61,62^

The portrayal of NSSI in the media and the ensuing effects of contagion are also elements likely contributing to the increased prevalence of NSSI among female adolescents across the globe. NSSI is often portrayed through a young female character in psychological distress who uses self-injury as a coping mechanism.^63^ Given the influential role of media and popular culture in the day-to-day life of adolescents and how media cross-permeates different cultures, it may be that female adolescents who identify with characters who engage in self-injurious behavior are more likely to engage in NSSI regardless of where they live.^63,64^

Although we found NSSI to be generally more common among female compared with male adolescents, there were some notable geographical exceptions. In Asia, the prevalence of NSSI was either equivalent in female and male adolescents, or the female-to-male ratio was reversed compared with other regions. A parallel trend in Asian populations is suicide rates by sex that are incongruent with the commonly reported gender paradox of suicide—more suicide attempts in female adolescents and more deaths by suicide in male adolescents^65^—seen in Western countries.^66,67^ These contrasting findings demonstrate the need to explore regional factors influencing sex differences in the prevalence of all suicide-related behaviors and not solely NSSI.

Notably, post-hoc analyses did not reveal a significant difference in the regional prevalence of NSSI among female adolescents but did reveal a higher prevalence of NSSI among male adolescents in Asia as compared with male adolescents in other regions. Our findings appear to suggest that female adolescents engage in equivalent rates of NSSI regardless of the region, whereas male adolescents in Asia engage in NSSI more often than in other regions. The reasons for this are unclear, and research is lacking, but it may be attributable to different gender roles and/or sociocultural factors in Asia vs Western cultures such as North America, Europe, and Australia.^68^ For example, Western media presents self-harm as a stereotypically female-gendered behavior^69^; hence, male adolescents living in these regions of the world may be less socialized than female adolescents to engage in NSSI. Previous research also demonstrates that NSSI may be used more commonly in non-Western countries as an attempt to regulate negative emotion specifically arising from interpersonal conflict, rather than to cope with emotion dysregulation more generally.^19,70,71^ Why this would be more relevant for male adolescents living in Asia warrants further examination. Lastly, it is possible that regionally rooted sociocultural factors may have affected participants’ willingness to self-report NSSI, depending on the stigma and/or perceived consequences of disclosing NSSI.^72^ These factors may have influenced underreporting of NSSI in certain groups, thus contributing to the geographical variations of NSSI prevalence by sex reported in this study. Given the regional variation of NSSI in male adolescents, there is a strong argument for further research to elucidate possible reasons for this and to allow for the development of regionally and perhaps culturally specific interventions for the treatment and prevention of NSSI among adolescents.

### Limitations

Limitations of this meta-analysis include the significant heterogeneity of study designs and sample characteristics (eg, age ranges) and that not all possible moderators or combinations of moderators could be accounted for. Furthermore, while all studies defined NSSI as self-injurious behavior without suicidal intent, participants were classified into an NSSI group based on varying criteria (eg, past month engagement in NSSI vs lifetime engagement in NSSI vs a certain frequency of NSSI in the past year). At minimum, participants classified as having engaged in NSSI would have done so at least once in their lifetime, but some studies employed more stringent criteria, making it difficult to directly compare findings between studies. It should also be noted that the majority of studies included in this analysis were conducted in upper-middle income and high-income countries across the globe, with limited numbers of samples from certain regions such as Australia and an absence of representation from South America or Africa, and thus these findings may not be able to be extrapolated to countries not represented here. The restriction of our search to English language articles is also a limitation given our focus on geographic variability. Lastly, most studies used participants’ self-reported sex and may not have accounted for cisgender vs transgender identity, limiting our ability to make any conclusions regarding the effect of gender identity vs sex assigned at birth. Given that gender dysphoria is a known risk factor for NSSI and suicide,^73^ future work in this area, with a focus on sex differences and regional variation, will be of paramount importance.

## Conclusions

In summary, this meta-analysis provides the important global and regional estimates of the female-to-male prevalence of NSSI among adolescents. Globally, NSSI was more prevalent among female adolescents than male adolescents, with equivalent prevalence estimates among female adolescents across regions. By contrast, the prevalence of NSSI among male adolescents was higher than that in female adolescents in Asia and male adolescents in other regions. Future research with sex-, region-, and culture-specific lenses will be essential in clarifying how and why NSSI phenotypes differ by sex in different regions. Research in this area will be foundational in developing and evaluating effective interventions for all adolescents engaged in NSSI regardless of location and sex.

## Notes

Supplement 1. **eMethods. **Full OVID Search Strategy **eFigure 1. **Moderator Analysis for Year of Publication Mean-Centred Around 2015 **eFigure 2. **Moderator Analysis for Asia (14 Samples) as Compared With Other Geographical Regions **eFigure 3. **Moderator Analysis for Europe (Samples) as Compared With Other Geographical Regions **eFigure 4.** Moderator Analysis for Lifetime NSSI vs Recent NSSI (Past Month to Past 5 Years) as Survey Measure **eFigure 5. **Funnel Plot of Effect Size (Log or Female:Male) vs SE to Assess for Publication Bias **eTable 1. **Characteristics of Included Studies **eTable 2. **ANOVA and t-Test Values for Post-Hoc Comparisons
eReferences.

Supplement 2.
Data Sharing Statement

## References

[1] Swannell SV, Martin GE, Page A, Hasking P, St John NJ. Prevalence of nonsuicidal self-injury in nonclinical samples: systematic review, meta-analysis and meta-regression. Suicide Life Threat Behav. 2014;44(3):273-303. doi:10.1111/sltb.12070 24422986

[2] Gillies D, Christou MA, Dixon AC, . Prevalence and characteristics of self-harm in adolescents: meta-analyses of community-based studies 1990-2015. J Am Acad Child Adolesc Psychiatry. 2018;57(10):733-741. doi:10.1016/j.jaac.2018.06.018 30274648

[3] Steinhoff A, Ribeaud D, Kupferschmid S, . Self-injury from early adolescence to early adulthood: age-related course, recurrence, and services use in males and females from the community. Eur Child Adolesc Psychiatry. 2021;30(6):937-951. doi:10.1007/s00787-020-01573-w 32572615 PMC8140957

[4] Albores-Gallo L, Méndez-Santos JL, Xóchitl-García Luna A, Delgadillo-González Y, Chávez-Flores CI, Martínez OL. Nonsuicidal self-injury in a community sample of older children and adolescents of Mexico City. Actas Esp Psiquiatr. 2014;42(4):159-168. 25017493

[5] You J, Leung F, Fu K, Lai CM. The prevalence of nonsuicidal self-injury and different subgroups of self-injurers in Chinese adolescents. Arch Suicide Res. 2011;15(1):75-86. doi:10.1080/13811118.2011.540211 21294002

[6] Muehlenkamp JJ, Claes L, Havertape L, Plener PL. International prevalence of adolescent non-suicidal self-injury and deliberate self-harm. Child Adolesc Psychiatry Ment Health. 2012;6(1):10. doi:10.1186/1753-2000-6-10 22462815 PMC3348041

[7] Kapur N, Cooper J, O’Connor RC, Hawton K. Non-suicidal self-injury v. attempted suicide: new diagnosis or false dichotomy? Br J Psychiatry. 2013;202(5):326-328. doi:10.1192/bjp.bp.112.116111 23637107

[8] Rhodes AE, Sinyor M, Boyle MH, . Emergency department presentations and youth suicide: a case-control study. Can J Psychiatry. 2019;64(2):88-97. doi:10.1177/0706743718802799 30282479 PMC6405805

[9] Whitlock J, Knox KL. The relationship between self-injurious behavior and suicide in a young adult population. Arch Pediatr Adolesc Med. 2007;161(7):634-640. doi:10.1001/archpedi.161.7.634 17606825

[10] Klonsky ED, May AM, Glenn CR. The relationship between nonsuicidal self-injury and attempted suicide: converging evidence from four samples. J Abnorm Psychol. 2013;122(1):231-237. doi:10.1037/a0030278 23067259

[11] Asarnow JR, Porta G, Spirito A, . Suicide attempts and nonsuicidal self-injury in the treatment of resistant depression in adolescents: findings from the TORDIA study. J Am Acad Child Adolesc Psychiatry. 2011;50(8):772-781. doi:10.1016/j.jaac.2011.04.003 21784297 PMC3143365

[12] Wilkinson P, Kelvin R, Roberts C, Dubicka B, Goodyer I. Clinical and psychosocial predictors of suicide attempts and nonsuicidal self-injury in the Adolescent Depression Antidepressants and Psychotherapy Trial (ADAPT). Am J Psychiatry. 2011;168(5):495-501. doi:10.1176/appi.ajp.2010.10050718 21285141

[13] Zetterqvist M, Lundh LG, Dahlström O, Svedin CG. Prevalence and function of non-suicidal self-injury (NSSI) in a community sample of adolescents, using suggested DSM-5 criteria for a potential NSSI disorder. J Abnorm Child Psychol. 2013;41(5):759-773. doi:10.1007/s10802-013-9712-5 23344701

[14] Sornberger MJ, Heath NL, Toste JR, McLouth R. Nonsuicidal self-injury and gender: patterns of prevalence, methods, and locations among adolescents. Suicide Life Threat Behav. 2012;42(3):266-278. doi:10.1111/j.1943-278X.2012.0088.x 22435988

[15] Sevecke K, Bock A, Fenzel L, Gander M, Fuchs M. Nonsuicidal self-injury in a naturalistic sample of adolescents undergoing inpatient psychiatric treatment: prevalence, gender distribution and comorbidities. Psychiatr Danub. 2017;29(4):522-528. doi:10.24869/psyd.2017.522 29197214

[16] Posporelis S, Paspali A, Takayanagi Y, Sawa A, Banerjea P, Kyriakopoulos M. Demographic and clinical correlates of suicidality in adolescents attending a specialist community mental health service: a naturalistic study. J Ment Health. 2015;24(4):225-229. doi:10.3109/09638237.2015.1022249 26203534

[17] Barrocas AL, Hankin BL, Young JF, Abela JRZ. Rates of nonsuicidal self-injury in youth: age, sex, and behavioral methods in a community sample. Pediatrics. 2012;130(1):39-45. doi:10.1542/peds.2011-2094 22689875 PMC3382916

[18] Howe-Martin LS, Murrell AR, Guarnaccia CA. Repetitive nonsuicidal self-injury as experiential avoidance among a community sample of adolescents. J Clin Psychol. 2012;68(7):809-829. doi:10.1002/jclp.21868 22589002

[19] You J, Leung F, Fu K. Exploring the reciprocal relations between nonsuicidal self-injury, negative emotions and relationship problems in Chinese adolescents: a longitudinal cross-lag study. J Abnorm Child Psychol. 2012;40(5):829-836. doi:10.1007/s10802-011-9597-0 22116636

[20] Tang J, Ma Y, Lewis SP, . Association of internet addiction with nonsuicidal self-injury among adolescents in China. JAMA Netw Open. 2020;3(6):e206863. doi:10.1001/jamanetworkopen.2020.6863 32496567 PMC7273191

[21] Monto MA, McRee N, Deryck FS. Nonsuicidal self-injury among a representative sample of US adolescents, 2015. Am J Public Health. 2018;108(8):1042-1048. doi:10.2105/AJPH.2018.304470 29927642 PMC6050840

[22] Kang N, Jiang Y, Ren Y, . Distress intolerance mediates the relationship between child maltreatment and nonsuicidal self-injury among Chinese adolescents: a three-wave longitudinal study. J Youth Adolesc. 2018;47(10):2220-2230. doi:10.1007/s10964-018-0877-7 29942987

[23] Cwik MF, Rosenstock S, Tingey L, . Characteristics of substance abuse and self-injury among American Indian adolescents who have engaged in binge drinking. Am Indian Alsk Native Ment Health Res. 2018;25(2):1-19. doi:10.5820/aian.2502.2018.1 29889946

[24] Ren Y, Lin MP, Liu YH, . The mediating role of coping strategy in the association between family functioning and nonsuicidal self-injury among Taiwanese adolescents. J Clin Psychol. 2018;74(7):1246-1257. doi:10.1002/jclp.22587 29355974

[25] DeCamp W, Bakken NW. Self-injury, suicide ideation, and sexual orientation: differences in causes and correlates among high school students. J Inj Violence Res. 2016;8(1):15-24. doi:10.5249/jivr.v8i1.545 26401756 PMC4729330

[26] Tseng FY, Yang HJ. Internet use and web communication networks, sources of social support, and forms of suicidal and nonsuicidal self-injury among adolescents: different patterns between genders. Suicide Life Threat Behav. 2015;45(2):178-191. doi:10.1111/sltb.12124 25255896

[27] Chang SS, Chen YY, Heron J, Kidger J, Lewis G, Gunnell D. IQ and adolescent self-harm behaviours in the ALSPAC birth cohort. J Affect Disord. 2014;152-154:175-182. doi:10.1016/j.jad.2013.09.005 24080206

[28] Prinstein MJ, Heilbron N, Guerry JD, . Peer influence and nonsuicidal self injury: longitudinal results in community and clinically-referred adolescent samples. J Abnorm Child Psychol. 2010;38(5):669-682. doi:10.1007/s10802-010-9423-0 20437255 PMC3686282

[29] Isohookana R, Riala K, Hakko H, Räsänen P. Adverse childhood experiences and suicidal behavior of adolescent psychiatric inpatients. Eur Child Adolesc Psychiatry. 2013;22(1):13-22. doi:10.1007/s00787-012-0311-8 22842795

[30] Bakken NW, Gunter WD. Self-cutting and suicidal ideation among adolescents: gender differences in the causes and correlates of self-injury. Deviant Behav. 2012;33:339-356. doi:10.1080/01639625.2011.584054

[31] Gandhi A, Luyckx K, Maitra S, Claes L. Non-suicidal self-injury and identity distress in Flemish adolescents: exploring gender differences and mediational pathways. Pers Individ Dif. 2015;82:215-220. doi:10.1016/j.paid.2015.03.031

[32] Xavier A, Cunha M, Pinto-Gouveia J. Daily peer hassles and non-suicidal self-injury in adolescence: gender differences in avoidance-focused emotion regulation processes. J Child Fam Stud. 2018;27(1):59-68. doi:10.1007/s10826-017-0871-9

[33] Laye-Gindhu A, Schonert-Reichl KA. Nonsuicidal self-harm among community adolescents: understanding the “whats” and “whys” of self-harm. J Youth Adolesc. 2005;34(5):447-457. doi:10.1007/s10964-005-7262-z

[34] Salmon S, Garcés Dávila I, Taillieu TL, . Adolescent health outcomes: associations with child maltreatment and peer victimization. BMC Public Health. 2022;22(1):905. doi:10.1186/s12889-022-13310-w 35524250 PMC9074223

[35] Jeong JY, Kim DH. Gender differences in the prevalence of and factors related to non-suicidal self-injury among middle and high school students in South Korea. Int J Environ Res Public Health. 2021;18(11):5965. doi:10.3390/ijerph18115965 34199442 PMC8199576

[36] Taliaferro LA, Muehlenkamp JJ. Risk and protective factors that distinguish adolescents who attempt suicide from those who only consider suicide in the past year. Suicide Life Threat Behav. 2014;44(1):6-22. doi:10.1111/sltb.12046 23855367

[37] Wan YH, Xu SJ, Chen J, Hu CL, Tao FB. Longitudinal effects of psychological symptoms on non-suicidal self-injury: a difference between adolescents and young adults in China. Soc Psychiatry Psychiatr Epidemiol. 2015;50(2):237-247. doi:10.1007/s00127-014-0917-x 24974078

[38] Xin M, Yang X, Liu K, Naz Boke B, Bastien L. Impact of negative life events and social support on nonsuicidal self-injury among chinese middle school students. Am J Mens Health. 2020;14(4):1557988320937124. Published online July 23, 2020. doi:10.1177/1557988320937124 32703057 PMC7383706

[39] Liu RT, Walsh RFL, Sheehan AE, Cheek SM, Sanzari CM. Prevalence and correlates of suicide and nonsuicidal self-injury in children: a systematic review and meta-analysis. JAMA Psychiatry. 2022;79(7):718-726. doi:10.1001/jamapsychiatry.2022.1256 35612875 PMC9134039

[40] Oktan V. A characterization of self-injurious behavior among Turkish adolescents. Psychol Rep. 2014;115(3):645-654. doi:10.2466/16.02.PR0.115c25z5 25350208

[41] Hanania JW, Heath NL, Emery AA, Toste JR, Daoud FA. Non-suicidal self-injury among adolescents in Amman, Jordan. Arch Suicide Res. 2015;19(2):260-274. doi:10.1080/13811118.2014.915778 25058810

[42] Wan Y, Chen R, Ma S, . Associations of adverse childhood experiences and social support with self-injurious behaviour and suicidality in adolescents. Br J Psychiatry. 2019;214(3):146-152. doi:10.1192/bjp.2018.263 30477603 PMC6429251

[43] Lee JY, Kim H, Kim SY, Kim JM, Shin IS, Kim SW. Non-suicidal self-injury is associated with psychotic like experiences, depression, and bullying in Korean adolescents. Early Interv Psychiatry. 2021;15(6):1696-1704. doi:10.1111/eip.13115 33461244

[44] Poudel A, Lamichhane A, Magar KR, Khanal GP. Non suicidal self injury and suicidal behavior among adolescents: co-occurrence and associated risk factors. BMC Psychiatry. 2022;22(1):96. doi:10.1186/s12888-022-03763-z 35139825 PMC8827284

[45] Wan Y, Chen R, Wang S, . Associations of coping styles with nonsuicidal self-injury in adolescents: do they vary with gender and adverse childhood experiences? Child Abuse Negl. 2020;104:104470. doi:10.1016/j.chiabu.2020.104470 32234639

[46] Cipriano A, Cella S, Cotrufo P. Nonsuicidal self-injury: a systematic review. Front Psychol. 2017;8:1946. doi:10.3389/fpsyg.2017.01946 29167651 PMC5682335

[47] US Centers for Disease Control and Prevention. Injury prevention and control: WISQARS - web-based injury statistics query reporting system. 2020. Accessed May 8, 2024. https://www.cdc.gov/injury/wisqars

[48] Gardner W, Pajer K, Cloutier P, . Changing rates of self-harm and mental disorders by sex in youths presenting to Ontario emergency departments: repeated cross-sectional study. Can J Psychiatry. 2019;64(11):789-797. doi:10.1177/0706743719854070 31184929 PMC6882075

[49] Page MJ, McKenzie JE, Bossuyt PM, . The PRISMA 2020 statement: an updated guideline for reporting systematic reviews. BMJ. 2021;372(71):n71. doi:10.1136/bmj.n71 33782057 PMC8005924

[50] Gratz KL. Measurement of deliberate self-harm: preliminary data on the Deliberate Self-Harm Inventory. J Psychopathol Behav Assess. 2001;23:253-263. doi:10.1023/A:1012779403943

[51] Joanna Briggs Institute. Critical appraisal checklist for analytical cross sectional studies. 2020. Accessed May 8, 2024. https://jbi.global/sites/default/files/2019-05/JBI_Critical_Appraisal-Checklist_for_Analytical_Cross_Sectional_Studies2017_0.pdf

[52] Joanna Briggs Institute. Critical appraisal checklist for cohort studies. 2020. Accessed May 8, 2024. https://jbi.global/sites/default/files/2019-05/JBI_Critical_Appraisal-Checklist_for_Cohort_Studies2017_0.pdf

[53] Suurmond R, van Rhee H, Hak T. Introduction, comparison, and validation of Meta-Essentials: a free and simple tool for meta-analysis. Res Synth Methods. 2017;8(4):537-553. doi:10.1002/jrsm.1260 28801932 PMC5725669

[54] Taliaferro LA, Muehlenkamp JJ. Factors associated with current versus lifetime self-injury among high school and college students. Suicide Life Threat Behav. 2015;45(1):84-97. doi:10.1111/sltb.12117 25169623

[55] Larsson B, Sund AM. Prevalence, course, incidence, and 1-year prediction of deliberate self-harm and suicide attempts in early Norwegian school adolescents. Suicide Life Threat Behav. 2008;38(2):152-165. doi:10.1521/suli.2008.38.2.152 18444774

[56] Martin G, Bergen HA, Richardson AS, Roeger L, Allison S. Sexual abuse and suicidality: gender differences in a large community sample of adolescents. Child Abuse Negl. 2004;28(5):491-503. doi:10.1016/j.chiabu.2003.08.006 15159067

[57] Baetens I, Claes L, Muehlenkamp J, Grietens H, Onghena P. Non-suicidal and suicidal self-injurious behavior among Flemish adolescents: a web-survey. Arch Suicide Res. 2011;15(1):56-67. doi:10.1080/13811118.2011.540467 21294000

[58] Wilkinson PO, Qiu T, Jesmont C, . Age and gender effects on non-suicidal self-injury, and their interplay with psychological distress. J Affect Disord. 2022;306:240-245. doi:10.1016/j.jad.2022.03.021 35304237

[59] Stewart SL, Baiden P, Theall-Honey L. Examining non-suicidal self-injury among adolescents with mental health needs, in Ontario, Canada. Arch Suicide Res. 2014;18(4):392-409. 24712902 10.1080/13811118.2013.824838

[60] Chen H, Cohen P, Chen S. How big is a big odds ratio? interpreting the magnitudes of odds ratios in epidemiological studies. Commun Stat Simul Comput. 2010;39(4):860-864. doi:10.1080/03610911003650383

[61] Van Droogenbroeck F, Spruyt B, Keppens G. Gender differences in mental health problems among adolescents and the role of social support: results from the Belgian health interview surveys 2008 and 2013. BMC Psychiatry. 2018;18(1):6. doi:10.1186/s12888-018-1591-4 29320999 PMC5763832

[62] Gutman LM, Codiroli McMaster N. Gendered pathways of internalizing problems from early childhood to adolescence and associated adolescent outcomes. J Abnorm Child Psychol. 2020;48(5):703-718. doi:10.1007/s10802-020-00623-w 32040796 PMC7188729

[63] Trewavas C, Hasking P, McAllister M. Representations of non-suicidal self-injury in motion pictures. Arch Suicide Res. 2010;14(1):89-103. doi:10.1080/13811110903479110 20112147

[64] Jamieson PE, Romer D. Trends in explicit portrayal of suicidal behavior in popular U.S. movies, 1950-2006. Arch Suicide Res. 2011;15(3):277-289. doi:10.1080/13811118.2011.589748 21827317

[65] Canetto SS, Sakinofsky I. The gender paradox in suicide. Suicide Life Threat Behav. 1998;28(1):1-23. doi:10.1111/j.1943-278X.1998.tb00622.x 9560163

[66] Chen YY, Wu KC, Yousuf S, Yip PSF. Suicide in Asia: opportunities and challenges. Epidemiol Rev. 2012;34(1):129-144. doi:10.1093/epirev/mxr025 22158651

[67] Wasserman D, Cheng Q, Jiang GX. Global suicide rates among young people aged 15-19. World Psychiatry. 2005;4(2):114-120. 16633527 PMC1414751

[68] Snowdon J. Differences between patterns of suicide in East Asia and the West. The importance of sociocultural factors. Asian J Psychiatr. 2018;37:106-111. doi:10.1016/j.ajp.2018.08.019 30173014

[69] Whitlock J, Knox KL. Intervention and Prevention in the Community. In: Nixon MK, Heath NL, eds. Self-Injury in Youth: The Essential Guide to Assessment and Intervention. 1st ed. Routledge; 2008:368.

[70] Chang SS, Steeg S, Kapur N, Webb RT, Yip PS, Cooper J. Self-harm amongst people of Chinese origin versus White people living in England: a cohort study. BMC Psychiatry. 2015;15(1):79. doi:10.1186/s12888-015-0467-0 25880647 PMC4409751

[71] Gholamrezaei M, De Stefano J, Heath NL. Nonsuicidal self-injury across cultures and ethnic and racial minorities: a review. Int J Psychol. 2017;52(4):316-326. doi:10.1002/ijop.12230 26644040

[72] Aggarwal S, Borschmann R, Patton GC. Tackling stigma in self-harm and suicide in the young. Lancet Public Health. 2021;6(1):e6-e7. doi:10.1016/S2468-2667(20)30259-0 33417848 PMC7611270

[73] Veale JF, Watson RJ, Peter T, Saewyc EM. Mental health disparities among canadian transgender youth. J Adolesc Health. 2017;60(1):44-49. doi:10.1016/j.jadohealth.2016.09.014 28007056 PMC5630273

