# Supplementary material for: Sex Differences in the Global Prevalence of Nonsuicidal Self-Injury in Adolescents: A Meta-Analysis
Source: JAMA Netw Open. 2024 Jun 14;7(6):e2415436. doi: 10.1001/jamanetworkopen.2024.15436 (PMC11179134; doi:10.1001/jamanetworkopen.2024.15436)
Supplement: Supplement 1. — eMethods. Full OVID Search Strategy eFigure 1. Moderator Analysis for Year of Publication Mean-Centred Around 2015 eFigure 2. Moderator Analysis for Asia (14 Samples) as Compared With Other Geographical Regions eFigure 3. Moderator Analysis for Europe (Samples) as Compared With Other Geographical Regions eFigure 4. Moderator Analysis for Lifetime NSSI vs Recent NSSI (Past Month to Past 5 Years) as Survey Measure eFigure 5. Funnel Plot of Effect Size (Log or Female:Male) vs SE to Assess for Publication Bias eTable 1. Characteristics of Included Studies eTable 2. ANOVA and t-Test Values for Post-Hoc Comparisons eReferences. [file jamanetwopen-e2415436-s001.pdf]

Supplemental Online Content

Moloney F, Amini J, Sinyor M, Schaffer A, Lanctot K, Mitchell R. Sex differences in the global prevalence of nonsuicidal self-injury in adolescents. *JAMA Netw Open*. 2024;7(6):e2415436.
doi:10.1001/jamanetworkopen.2024.15436

eMethods. Full OVID Search Strategy
eFigure 1. Moderator Analysis for Year of Publication Mean-Centred Around 2015
eFigure 2. Moderator Analysis for Asia (14 Samples) as Compared With Other Geographical Regions
eFigure 3. Moderator Analysis for Europe (8 Samples) as Compared With Other Geographical Regions
eFigure 4. Moderator Analysis for Lifetime NSSI vs Recent NSSI (Past Month to Past 5 Years) as Survey Measure
eFigure 5. Funnel Plot of Effect Size (Log OR Female:Male) vs SE to Assess for Publication Bias
eTable 1. Characteristics of Included Studies
eTable 2. ANOVA and t-Test Values for Post-Hoc Comparisons
eReferences.

This supplemental material has been provided by the authors to give readers additional information about their work.

© 2024 Moloney F et al. *JAMA Network Open.*

eMethods. Full OVID Search Strategy

1. (teen* or youth* or adolescen* or juvenile* or girl* or boy* or (young adj2 (adult* or person* or individual* or people* or population* or man or men or wom#n)) or highschool* or ((secondary or high*) adj2 (school* or education))).tw,kf. or adolescent/ or young adult/
2. (parasuicid* or self-harm or self-injur* or self-mutilat* or self-poison* or ((self or themsel*) adj3 (cut or cutting or harm or poison* or hurt* or injur* or mutilate*))).tw,kf. or self-injurious behavior/ or self-mutilation/
3. (sex based or sex-based or sex factors or sex distribution or sex characteristics or gender difference* or gender based or gender-based or gender factor or (sex adj3 difference*) or (gender adj3 difference*)).tw,kf. or sex characteristics/ or sex factors/
4. 1 and 2 and 3

© 2024 Moloney F et al. *JAMA Network Open.*

eFigure 1. Moderator Analysis for Year of Publication Mean-Centred Around 2015

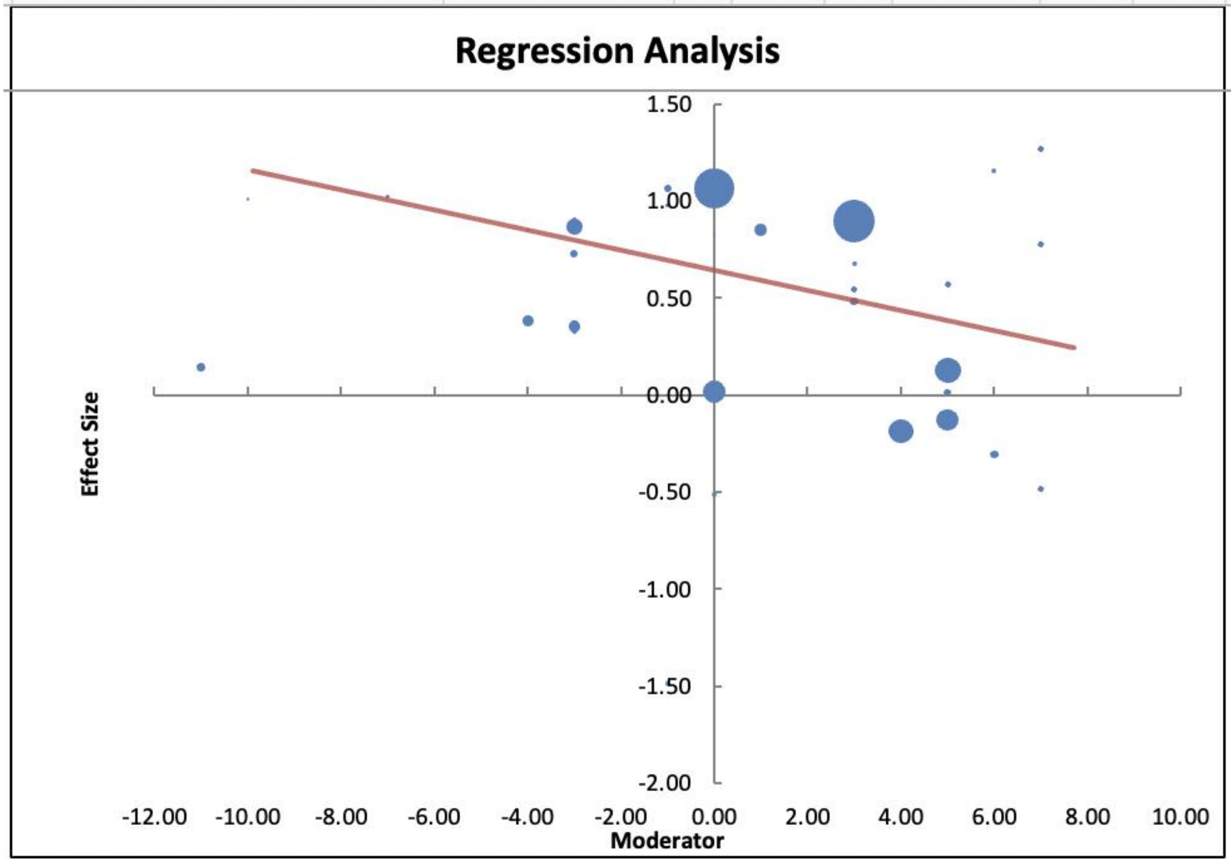

	B	SE	CI LL	CI UL	β	Z-value	p-value
Intercept	0.65	0.01	0.62	0.67		50.16	0.000
Moderator	-0.05	0.00	-0.06	-0.04	-0.30	-13.94	0.000

Analysis of variance	Sum of squares (Q)	df	p	Mean square	F-Value	p-value
Model	194.43	1	0.000	194.43	3.19	0.084
Residual	1952.59	32	0.000	61.02		
Total	2147.02	33	0.000			

Combined effect size	0.55
T^2 (method of moments estimation)	0.29
R^2	9.06%

© 2024 Moloney F et al. *JAMA Network Open.*

eFigure 2. Moderator Analysis for Asia (14 Samples) as Compared With Other Geographical Regions

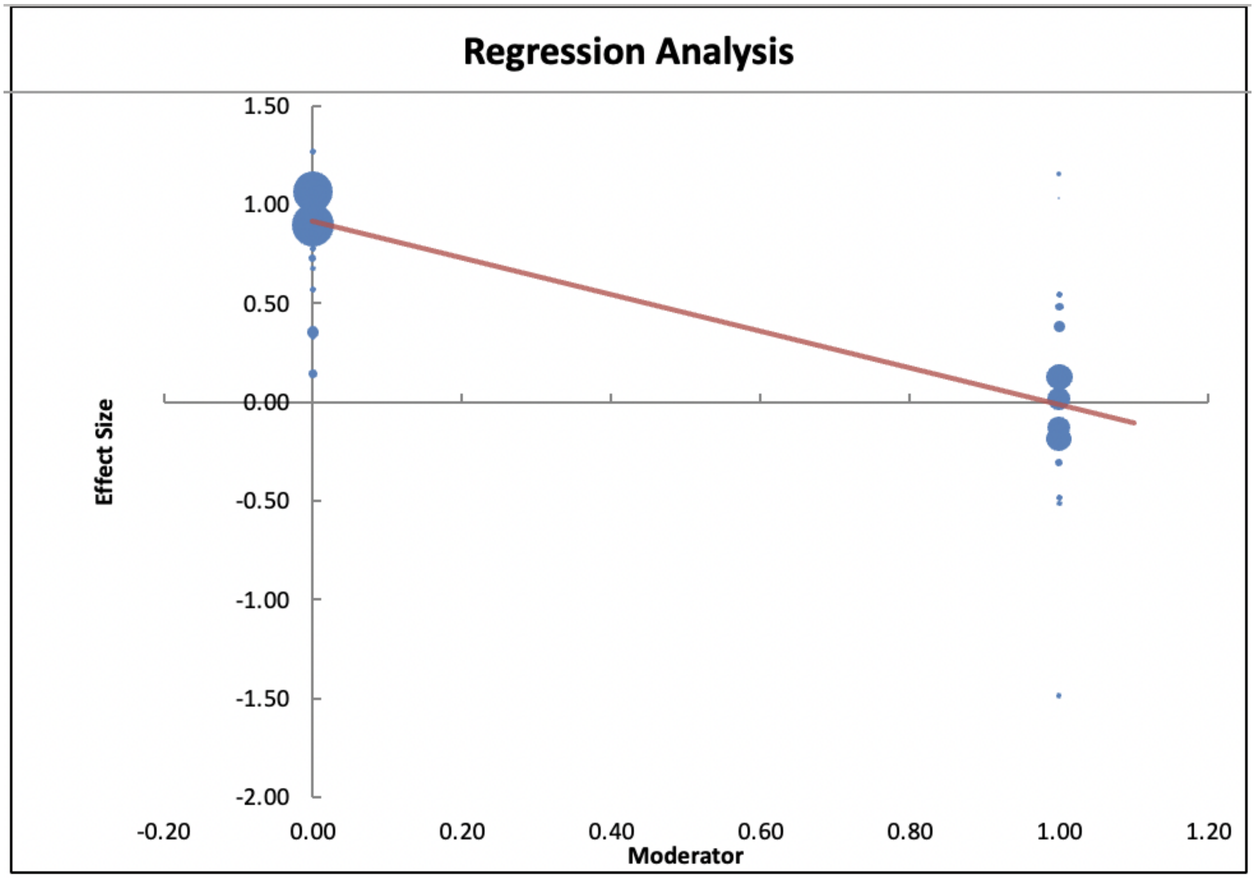

	B	SE	CI LL	CI UL	β	Z-value	p-value
Intercept	0.92	0.01	0.89	0.95		65.17	0.000
Moderator	-0.93	0.02	-0.98	-0.89	-0.90	-41.48	0.000

Analysis of variance	Sum of squares (Q)	df	p	Mean square	F-Value	p-value
Model	1720.78	1	0.000	1720.78	129.19	0.000
Residual	426.24	32	0.000	13.32		
Total	2147.02	33	0.000			

Combined effect size	0.55
T^2 (method of moments estimation)	0.06
R^2	80.15%

© 2024 Moloney F et al. *JAMA Network Open.*

eFigure 3. Moderator Analysis for Europe (8 Samples) as Compared With Other Geographical Regions

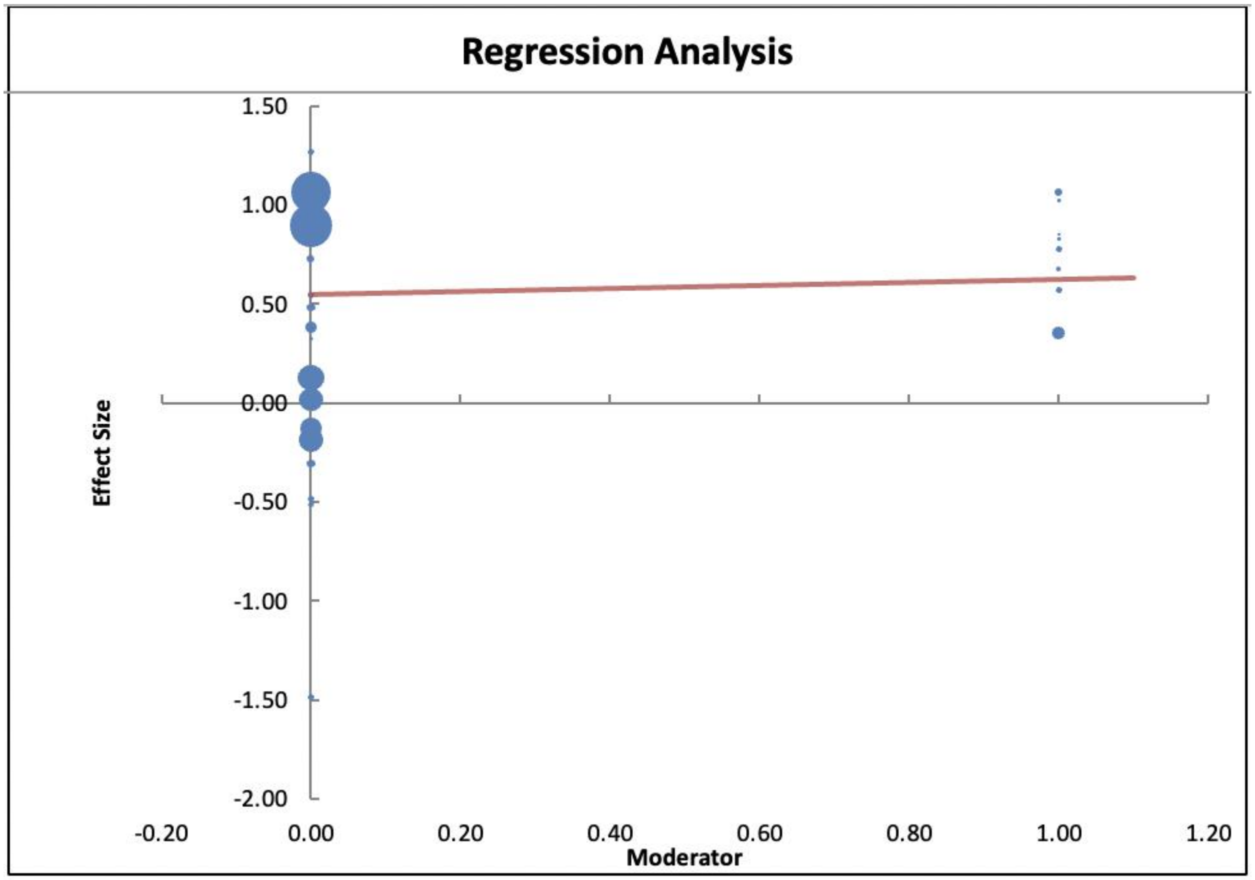

	B	SE	CI LL	CI UL	β	Z-value	p-value
Intercept	0.55	0.01	0.52	0.57		48.67	0.000
Moderator	0.08	0.05	-0.02	0.18	0.03	1.55	0.120

Analysis of variance	Sum of squares (Q)	df	p	Mean square	F-Value	p-value
Model	2.41	1	0.120	2.41	0.04	0.851
Residual	2144.61	32	0.000	67.02		
Total	2147.02	33	0.000			

Combined effect size	0.55
T^2 (method of moments estimation)	0.30
R^2	0.11%

© 2024 Moloney F et al. *JAMA Network Open.*

eFigure 4. Moderator Analysis for Lifetime NSSI vs Recent NSSI (Past Month to Past 5 Years) as Survey Measure

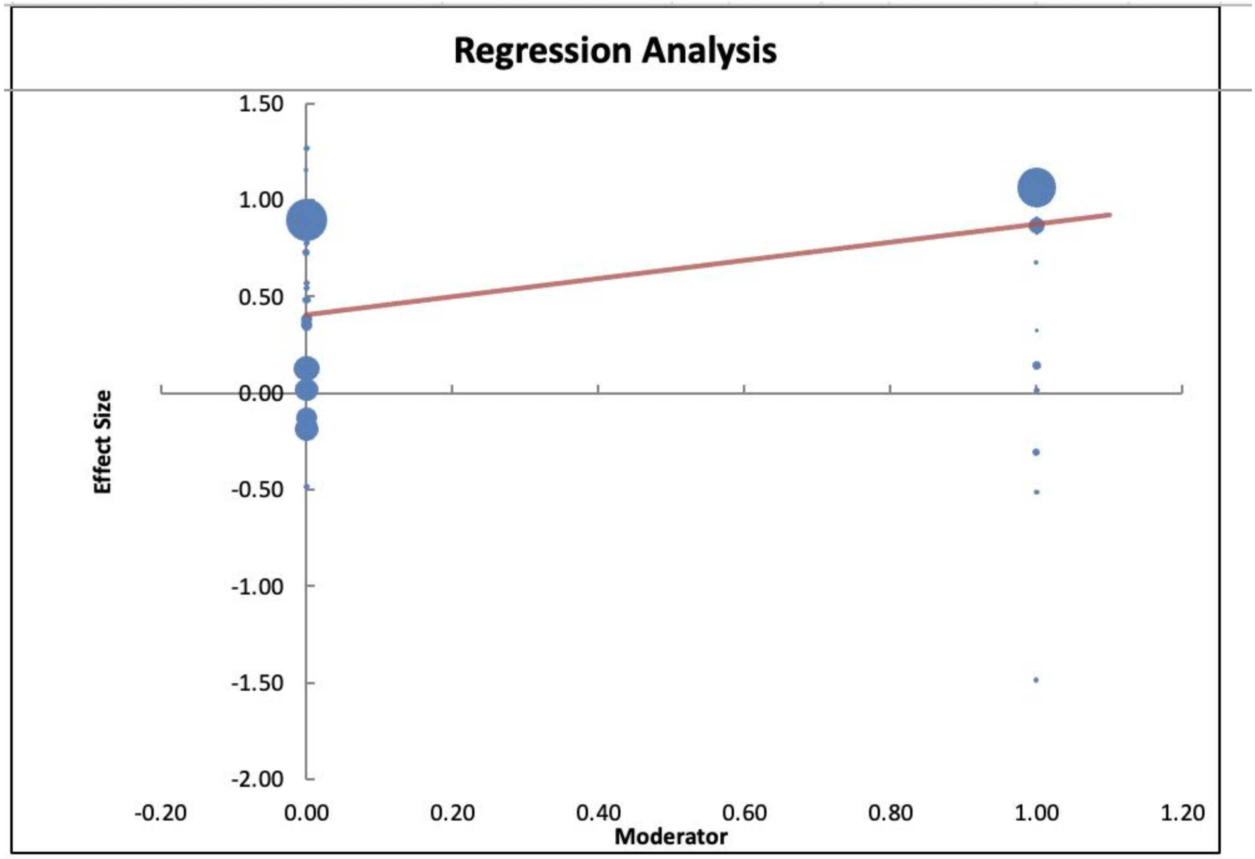

	B	SE	CI LL	CI UL	β	Z-value	p-value
Intercept	0.40	0.01	0.38	0.43		30.40	0.000
Moderator	0.48	0.02	0.43	0.52	0.43	20.12	0.000

Analysis of variance	Sum of squares (Q)	df	p
Model	404.75	1	0.000
Residual	1742.27	32	0.000
Total	2147.02	33	0.000

Mean square	F-Value	p-value
404.75	7.43	0.010
54.45		

Combined effect size	0.55
T^2 (method of moments estimation)	0.29
R^2	18.85%

© 2024 Moloney F et al. *JAMA Network Open.*

eFigure 5. Funnel Plot of Effect Size (Log or Female:Male) vs SE to Assess for Publication Bias

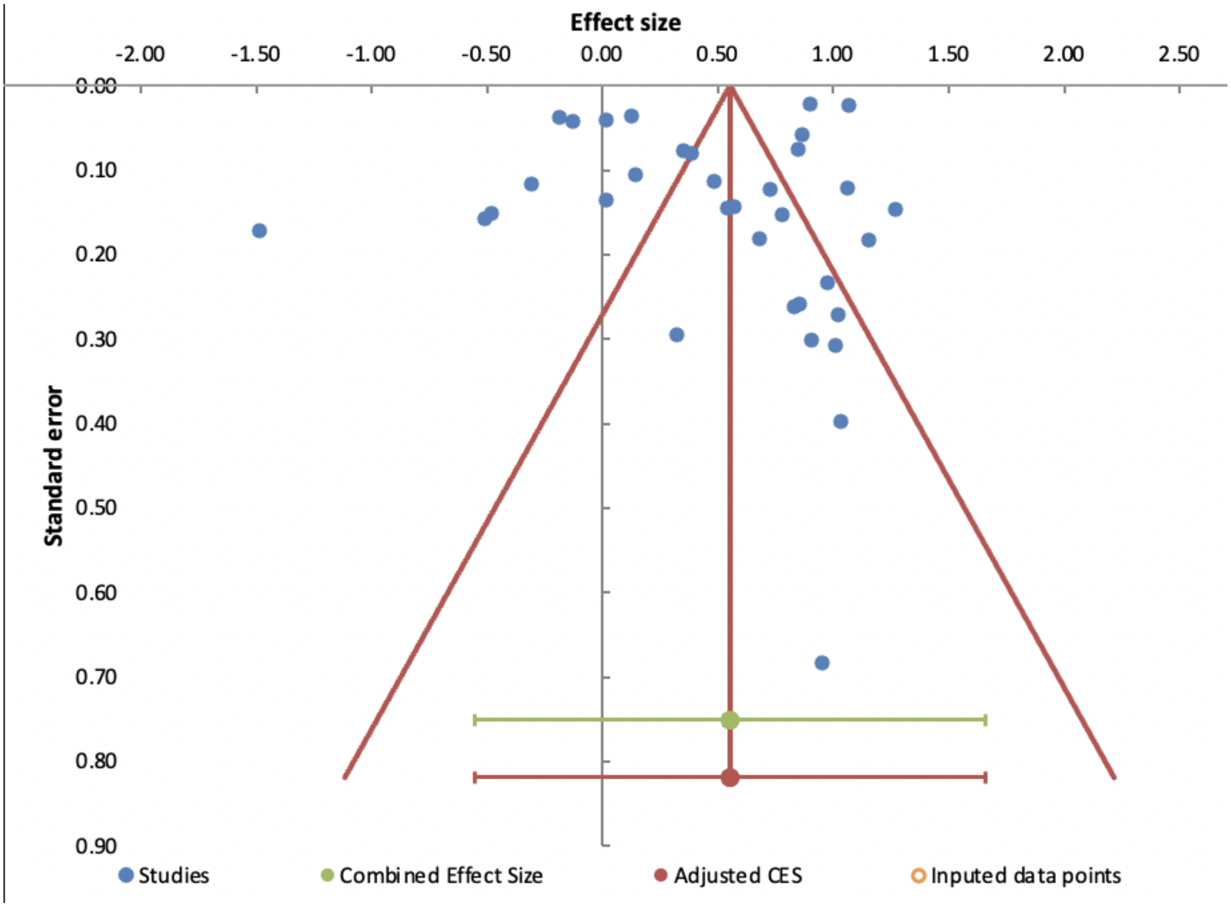

© 2024 Moloney F et al. *JAMA Network Open.*

eTable 1. Characteristics of Included Studies

Authors	Year	Country	Aims	Participants	Methods
Albores-Gallo *et al.*[1]	2014	Mexico	Describe frequency and characteristics of NSSI in adolescents	n = 533<br>Age 11-17<br>54% female<br>Community sample	Cross-sectional survey
Baetens *et al.*[2]	2011	Belgium	Investigate prevalence of NSSI and differences in psychological correlates between NSSI and SSI	n = 1417<br>Age 12-18<br>81.4% female<br>Community sample	Cross-sectional survey
Bakken and Gunter[3]	2012	US	Investigate gender differences in the causes and correlates of NSSI	n = 2639<br>Age 14-18<br>50% female<br>Community sample	Cross-sectional survey
Barrocas *et al.*[4]	2012	US	Assess the rate and methods of NSSI in adolescents	n = 655<br>Age 7-16<br>56% female<br>Community sample	Cross-sectional survey
Chang *et al.*[5]	2014	UK	Examine the relationship between IQ and suicide	n = 3560<br>Age 16-17<br>58% female<br>Community sample	Prospective cohort study<br>Participants followed from age 8 to 16/17 years
Cwik *et al.*[6]	2018	US	Determine the relationship between substance use and self-injury in American Indian adolescents who binge drink	n = 68<br>Age 10-19<br>44% female<br>Community sample	Cross-sectional survey
DeCamp and Bakken[7]	2016	US	Determine the prevalence of and risk factors for NSSI in sexual minority adolescents	n = 7326<br>High school age<br>49% female<br>Community sample	Cross-sectional survey
Gandhi *et al.*[8]	2015	Belgium	Explore associations between identity distress and NSSI	n = 568<br>Age 13-21<br>61.8% female<br>Community sample	Cross-sectional survey
Hanania *et al.*[9]	2015	Jordan	Examine the prevalence and characteristics of NSSI in adolescents in Jordan	n = 952<br>Age 11-19<br>49.8% female<br>Community sample	Cross-sectional survey
Howe-Martin *et al.*[10]	2012	US	Examine the relationship between experiential avoidance and NSSI in adolescents	n = 211<br>Age 13-18<br>50.7% female<br>Community sample	Cross-sectional survey

© 2024 Moloney F et al. *JAMA Network Open.*

Isohookana *et al.*[11]	2013	Finland	Examine the relationship between ACEs and suicidal behaviour in adolescents	n = 508<br>Age 12-17<br>59% female<br>Clinical sample (psychiatric inpatients)	Cross-sectional survey
Jeong and Kim.[12]	2021	South Korea	Estimate the prevalence of NSSI among adolescents and examine risk factors	n = 1674<br>Age 15-17<br>36.5% female<br>Community sample	Cross-sectional survey
Kang *et al.*[13]	2018	China	Determine the temporal relationship between maltreatment and NSSI with distress intolerance as a mediator	n = 3555<br>Age 12-19<br>52% female<br>Community sample	Prospective cohort study<br>0, 6, and 12 mo follow-up
Larsson and Sund[14]	2008	Norway	Examine the prevalence, course, and incidence of self-harm behaviours in adolescents	n = 2464<br>Age 12-15<br>50.8% female<br>Community sample	Prospective cohort study<br>0, and 1 year follow-up
Laye-Gindhu and Schonert-Reichl[15]	2005	Canada	Identify the prevalence and types of self-harm, elucidate the nature and underlying function of self-harm, and evaluate the relation of psychological adjustment, sociodemographic, and health-risk variables to self-harm in adolescents	n = 424<br>Age 13-18<br>55.7% female<br>Community sample	Cross-sectional survey
Lee *et al.*[16]	2021	South Korea	Examine associated characteristics predicting NSSI among adolescents	n = 1674<br>Age 15-17<br>36.5% female<br>Community sample	Cross-sectional survey
Martin *et al.*[17]	2004	Australia	Examine gender-specific relationships between self-reported sexual abuse in childhood and suicidality	n = 2485<br>Age 14<br>44% female<br>Community sample	Cross-sectional survey
Monto *et al.*[18]	2018	US	Describe prevalence of NSSI in community adolescents and identify sex differences in health risks associated with NSSI	n = 64 671<br>Age 14-18<br>50% female<br>Community sample	Cross-sectional survey
Oktan[19]	2014	Turkey	Assess type and frequency of NSSI in high school students in Turkey and identify sex differences	n = 680<br>Mean age 16.9<br>55% female<br>Community sample	Cross-sectional survey
Posporelis *et al.*[20]	2015	UK	Evaluate demographic and clinical correlates of self-harm and suicidality in	n = 149<br>Age 12-18<br>56.4% female<br>Clinical sample	Naturalistic study

© 2024 Moloney F et al. *JAMA Network Open.*

			adolescents	(community mental health service)	
Poudel *et al.*[21]	2022	Nepal	Determine the prevalence of and risk factors for NSSI and suicidal behaviours in adolescents in Nepal	n = 730<br>Age 12-19<br>54% female<br>Community sample	Cross-sectional survey
Prinstein *et al.*[22]	2010	US	Examine the impact of peer influence on NSSI in adolescents in the community and those receiving psychiatric services	n = 377 (community, 50% female) and n = 140 (clinical, 72% female)<br>Age 11-15	Prospective cohort study<br>0, and one year follow-up (community sample)<br>0, 9, and 18 mo follow-up (clinical sample)
Ren *et al.*[23]	2018	Taiwan	Examine the role of family functioning and coping strategies NSSI and assess gender differences in the findings	n = 1989<br>Mean age 15<br>52% female<br>Community sample	Cross-sectional survey
Salmon *et al.*[24]	2022	Canada	Determine the impact of child maltreatment and peer victimization on mental and physical health outcomes including NSSI	n = 2910<br>Age 14-17<br>48.6% female<br>Community sample	Cross-sectional survey
Sornberger *et al.*[25]	2012	US	Investigate differences in characteristics of NSSI between male and female adolescents	n = 7126<br>Age 11-19<br>50.8% female<br>Community sample	Cross-sectional survey
Steinhoff *et al.*[26]	2021	Switzerland	Describe prevalence, frequency, course, recurrence, and service use of NSSI in male and female adolescents	n = 1482<br>Age 7-20<br>48% female<br>Community sample	Prospective cohort study<br>NSSI assessed at age 13, 15, 17, and 20
Stewart *et al.*[27]	2014	Canada	Examine prevalence of and factors associated with NSSI in adolescents receiving mental health services	n = 2013<br>Age 14-18<br>44.5% female<br>Clinical sample	Retrospective chart review
Taliaferro and Muehlenkamp[28]	2015	US	Determine factors associated with lifetime vs current NSSI	n = 84121<br>Grades 9-12<br>50.3% female<br>Community sample	Cross-sectional survey
Tang *et al.*[29]	2020	China	Describe the relationship between internet addiction and NSSI	n = 15 623<br>Age 10-20<br>48.5% female<br>Community sample	Cross-sectional survey
Tseng and Yang[30]	2015	Taiwan	Determine the association between	n = 391<br>Age 13-18	Cross-sectional survey

© 2024 Moloney F et al. *JAMA Network Open.*

			internet use and self-injurious thoughts and behaviours in adolescents	55% female<br>Community sample	
Wan *et al.*[31]	2020	China	Identify gender differences in the impact of coping skills on NSSI	n = 9704<br>Age 11-19<br>52.6% female<br>Community sample	Cross-sectional survey
Wan *et al.*[32]	2019	China	Examine the effects of ACEs and social support on NSSI and suicidality and identify sex differences	n = 14 820<br>Age 10-20<br>50.2% female<br>Community sample	Cross-sectional survey
Wan *et al.*[33]	2015	China	Examine longitudinal relationship between psychological symptoms and NSSI in adolescents and young adults	n = 17 622<br>Age 12-24<br>51.2% female<br>Community sample	Prospective cohort<br>0, 3, 6, and 9 mo follow-up
Wilkinson *et al.*[34]	2022	UK	Examine the effects of age and gender on NSSI and their interplay with psychological distress	n = 2368<br>Age 14-15<br>54% female<br>Community sample	Cross-sectional survey
Xavier *et al.*[35]	2018	Portugal	Examine the mediating role of rumination, experiential avoidance, dissociation and depressive symptoms in the association between daily peer hassles and non-suicidal self-injury among adolescents	n = 776<br>Age 12-18<br>52.4% female<br>Community sample	Cross-sectional survey
Xin *et al.*[36]	2020	China	Describe the impact of negative life events and social support on NSSI in adolescents	n = 1180<br>Age 11-24<br>44% female<br>Community sample	Cross-sectional survey
You *et al.*[37]	2011	China	Investigate the prevalence of NSSI in Chinese adolescents and compare psychological correlates of different subgroups	n = 6374<br>Age 11-19<br>67.6% female<br>Community sample	Cross-sectional survey
Zetterqvist *et al.*[38]	2013	Sweden	Assess prevalence, characteristics, and functions of NSSI in adolescents	n = 3060<br>Age 15-17<br>50.5% female<br>Community sample	Cross-sectional survey

© 2024 Moloney F et al. *JAMA Network Open.*

eTable 2. ANOVA and *t*-Test Values for Post-Hoc Comparisons

Post-hoc comparisons	*F* or *t*-value
Female NSSI prevalence, whole sample (ANOVA)	F(2, 30) =.10
Male NSSI prevalence, whole sample (ANOVA)	F(2,30)= 5.31
Male NSSI prevalence, Asia to North America comparison (t-test)	t=3.2
Male NSSI prevalence, Asia to Europe comparison (t-test)	t=2.3
Male NSSI prevalence, Europe to North America comparison (t-test)	t=-0.4

© 2024 Moloney F et al. *JAMA Network Open.*

eReferences

1. Albores-Gallo, L. *et al.* Nonsuicidal self-injury in a community sample of older children and adolescents of Mexico City. *Actas Esp Psiquiatr* **42**, 159–168 (2014).
2. Baetens, I., Claes, L., Muehlenkamp, J., Grietens, H. & Onghena, P. Non-suicidal and suicidal self-injurious behavior among Flemish adolescents: A web-survey. *Arch Suicide Res* **15**, 56–67 (2011).
3. Bakken, N. W. & Gunter, W. D. Self-cutting and suicidal ideation among adolescents: Gender differences in the causes and correlates of self-injury. *Deviant Behavior* **33**, 339–356 (2012).
4. Barrocas, A. L., Hankin, B. L., Young, J. F. & Abela, J. R. Z. Rates of nonsuicidal self-injury in youth: age, sex, and behavioral methods in a community sample. *Pediatrics* **130**, 39–45 (2012).
5. Chang, S.-S. *et al.* IQ and adolescent self-harm behaviours in the ALSPAC birth cohort. *J Affect Disord* **152–154**, 175–182 (2014).
6. Cwik, M. F. *et al.* Characteristics of Substance Abuse and Self-Injury among American Indian Adolescents Who Have Engaged in Binge Drinking. *Am Indian Alsk Native Ment Health Res* **25**, 1–19 (2018).
7. DeCamp, W. & W.Bakken, N. Self-injury, suicide ideation, and sexual orientation: differences in causes and correlates among high school students. *J Inj Violence Res* **8**, 15–24 (2016).
8. Gandhi, A., Luyckx, K., Maitra, S. & Claes, L. Non-suicidal self-injury and identity distress in Flemish adolescents: Exploring gender differences and mediational pathways. *Personality and Individual Differences* **82**, 215–220 (2015).
9. Hanania, J. W., Heath, N. L., Emery, A. A., Toste, J. R. & Daoud, F. A. Non-Suicidal Self-Injury among Adolescents in Amman, Jordan. *Arch Suicide Res* **19**, 260–274 (2015).
10. Howe-Martin, L. S., Murrell, A. R. & Guarnaccia, C. A. Repetitive nonsuicidal self-injury as experiential avoidance among a community sample of adolescents. *J Clin Psychol* **68**, 809–829 (2012).
11. Isohookana, R., Riala, K., Hakko, H. & Räsänen, P. Adverse childhood experiences and suicidal behavior of adolescent psychiatric inpatients. *Eur Child Adolesc Psychiatry* **22**, 13–22 (2013).
12. Jeong, J.-Y. & Kim, D.-H. Gender Differences in the Prevalence of and Factors Related to Non-Suicidal Self-Injury among Middle and High School Students in South Korea. *Int J Environ Res Public Health* **18**, 5965 (2021).
13. Kang, N. *et al.* Distress Intolerance Mediates the Relationship between Child Maltreatment and Nonsuicidal Self-Injury among Chinese Adolescents: A Three-Wave Longitudinal Study. *J Youth Adolesc* **47**, 2220–2230 (2018).
14. Larsson, B. & Sund, A. M. Prevalence, course, incidence, and 1-year prediction of deliberate self-harm and suicide attempts in early Norwegian school adolescents. *Suicide Life Threat Behav* **38**, 152–165 (2008).
15. Laye-Gindhu, A. & Schonert-Reichl, K. A. Nonsuicidal Self-Harm Among Community Adolescents: Understanding the "Whats" and "Whys" of Self-Harm. *J Youth Adolescence* **34**, 447–457 (2005).
16. Lee, J.-Y. *et al.* Non-suicidal self-injury is associated with psychotic like experiences, depression, and bullying in Korean adolescents. *Early Interv Psychiatry* **15**, 1696–1704 (2021).
17. Martin, G., Bergen, H. A., Richardson, A. S., Roeger, L. & Allison, S. Sexual abuse and suicidality: gender differences in a large community sample of adolescents. *Child Abuse Negl* **28**, 491–503 (2004).
18. Monto, M. A., McRee, N. & Deryck, F. S. Nonsuicidal Self-Injury Among a Representative Sample of US Adolescents, 2015. *Am J Public Health* **108**, 1042–1048 (2018).
19. Oktan, V. A characterization of self-injurious behavior among Turkish adolescents. *Psychol Rep* **115**, 645–654 (2014).
20. Posporelis, S. *et al.* Demographic and clinical correlates of suicidality in adolescents attending a specialist community mental health service: a naturalistic study. *J Ment Health* **24**, 225–229 (2015).
21. Poudel, A., Lamichhane, A., Magar, K. R. & Khanal, G. P. Non suicidal self injury and suicidal behavior among adolescents: co-occurrence and associated risk factors. *BMC Psychiatry* **22**, 96 (2022).
22. Prinstein, M. J. *et al.* Peer influence and nonsuicidal self injury: longitudinal results in community and clinically-referred adolescent samples. *J Abnorm Child Psychol* **38**, 669–682 (2010).
23. Ren, Y. *et al.* The mediating role of coping strategy in the association between family functioning and nonsuicidal self-injury among Taiwanese adolescents. *J Clin Psychol* **74**, 1246–1257 (2018).
24. Salmon, S. *et al.* Adolescent health outcomes: associations with child maltreatment and peer victimization. *BMC Public Health* **22**, 905 (2022).
25. Sornberger, M. J., Heath, N. L., Toste, J. R. & McLouth, R. Nonsuicidal self-injury and gender: patterns of prevalence, methods, and locations among adolescents. *Suicide Life Threat Behav* **42**, 266–278 (2012).
26. Steinhoff, A. *et al.* Self-injury from early adolescence to early adulthood: age-related course, recurrence, and services use in males and females from the community. *Eur Child Adolesc Psychiatry* **30**, 937–951 (2021).
27. Stewart, S. L., Baiden, P. & Theall-Honey, L. Examining non-suicidal self-injury among adolescents with mental health needs, in Ontario, Canada. *Arch Suicide Res* **18**, 392–409 (2014).
28. Taliaferro, L. A. & Muehlenkamp, J. J. Factors associated with current versus lifetime self-injury among high school and college

students. *Suicide Life Threat Behav* **45**, 84–97 (2015).
29. Tang, J. *et al.* Association of Internet Addiction With Nonsuicidal Self-injury Among Adolescents in China. *JAMA Network Open* **3**, e206863 (2020).
30. Tseng, F.-Y. & Yang, H.-J. Internet use and web communication networks, sources of social support, and forms of suicidal and nonsuicidal self-injury among adolescents: different patterns between genders. *Suicide Life Threat Behav* **45**, 178–191 (2015).
31. Wan, Y. *et al.* Associations of coping styles with nonsuicidal self-injury in adolescents: Do they vary with gender and adverse childhood experiences? *Child Abuse Negl* **104**, 104470 (2020).
32. Wan, Y. *et al.* Associations of adverse childhood experiences and social support with self-injurious behaviour and suicidality in adolescents. *Br J Psychiatry* **214**, 146–152 (2019).
33. Wan, Y.-H., Xu, S.-J., Chen, J., Hu, C.-L. & Tao, F.-B. Longitudinal effects of psychological symptoms on non-suicidal self-injury: a difference between adolescents and young adults in China. *Soc Psychiatry Psychiatr Epidemiol* **50**, 237–247 (2015).
34. Wilkinson, P. O. *et al.* Age and gender effects on non-suicidal self-injury, and their interplay with psychological distress. *J Affect Disord* **306**, 240–245 (2022).
35. Xavier, A., Cunha, M. & Pinto-Gouveia, J. Daily Peer Hassles and Non-Suicidal Self-Injury in Adolescence: Gender Differences in Avoidance-Focused Emotion Regulation Processes. *J Child Fam Stud* **27**, 59–68 (2018).
36. Xin, M., Yang, X., Liu, K., Naz Boke, B. & Bastien, L. Impact of Negative Life Events and Social Support on Nonsuicidal Self-Injury Among Chinese Middle School Students. *Am J Mens Health* **14**, 1557988320937124 (2020).
37. You, J., Leung, F., Fu, K. & Lai, C. M. The prevalence of nonsuicidal self-injury and different subgroups of self-injurers in Chinese adolescents. *Arch Suicide Res* **15**, 75–86 (2011).
38. Zetterqvist, M., Lundh, L.-G., Dahlström, O. & Svedin, C. G. Prevalence and function of non-suicidal self-injury (NSSI) in a community sample of adolescents, using suggested DSM-5 criteria for a potential NSSI disorder. *J Abnorm Child Psychol* **41**, 759–773 (2013).

© 2024 Moloney F et al. *JAMA Network Open.*
