# Supplementary material for: Sex Differences in the Global Prevalence of Nonsuicidal Self-Injury in Adolescents: A Meta-Analysis
Source: JAMA Netw Open. 2024 Jun 14;7(6):e2415436. doi: 10.1001/jamanetworkopen.2024.15436 (PMC11179134; doi:10.1001/jamanetworkopen.2024.15436)
Supplement: Supplement 2. — Data Sharing Statement [file jamanetwopen-e2415436-s002.pdf]

Data Sharing Statement

Moloney. Sex Differences in the Global Prevalence of Nonsuicidal Self-Injury in Adolescents. *JAMA Netw Open*. Published June 14, 2024. doi:10.1001/jamanetworkopen.2024.15436

Data
Data available: Yes
Data types: Deidentified participant data
How to access data: All data are available in the Supplemental tables.
When available: With publication
